# Severe Acute Respiratory Syndrome Coronavirus 2 Viral RNA Load Status and Antibody Distribution Among Patients and Asymptomatic Carriers in Central China

**DOI:** 10.3389/fcimb.2021.559447

**Published:** 2021-03-19

**Authors:** Youhua Yuan, Huiling Wang, Jing Zhao, Nan Jing, Junhong Xu, Wei Li, Bing Ma, Jiangfeng Zhang, Gang Li, Shanmei Wang, Yi Li, Yuming Wang, Enguo Fan, Li Li

**Affiliations:** ^1^ Department of Clinical Microbiology, Henan Provincial People’s Hospital, People’s Hospital of Zhengzhou University, and People’s Hospital of Henan University, Zhengzhou, China; ^2^ Department of Polymerase Chain Reaction (PCR), Henan Provincial People’s Hospital, People’s Hospital of Zhengzhou University, and People’s Hospital of Henan University, Zhengzhou, China; ^3^ Department of Infectious Disease, Henan Provincial People’s Hospital, People’s Hospital of Zhengzhou University, and People’s Hospital of Henan University, Zhengzhou, China; ^4^ Department of Clinical Laboratory, Henan Provincial People’s Hospital, People’s Hospital of Zhengzhou University, and People’s Hospital of Henan University, Zhengzhou, China; ^5^ Department of Research Management, Henan Provincial People’s Hospital, People’s Hospital of Zhengzhou University, and People’s Hospital of Henan University, Zhengzhou, China

**Keywords:** severe acute respiratory syndrome coronavirus-2, coronavirus disease, viral RNA load, asymptomatic carriers, IgM, IgG

## Abstract

This study aimed to monitor severe acute respiratory syndrome coronavirus 2 (SARS-CoV-2) viral loads and specific serum-antibodies (immunoglobulin [Ig] G and M) among confirmed patients and asymptomatic carriers from returning healthy travelers. The throat swabs, sputum, and stool samples from 57 hospitalized coronavirus disease (COVID-19) patients and 8 asymptomatic carriers, among 170 returning healthy travelers were tested using reverse-transcription real-time polymerase chain reaction. SARS-CoV-2 IgM/IgG antibodies were detected *via* serum chemiluminescence assay. Sequential results showed higher viral RNA loads in the throat, sputum, and stool samples at 3–12 and 6–21 days after symptom onset among severely ill COVID-19 patients. Shorter viral habitation time (1–8 days) was observed in the oropharyngeal site and intestinal tract of asymptomatic carriers. The IgG and IgM response rates were 19/37 (51.4%) and 23/37 (62.6%) among the 29 confirmed patients and 8 asymptomatic carriers, respectively, within 66 days from symptom or detection onset. The median duration between symptom onset and positive IgG and IgM results was 30 (n=23; interquartile range [IQR]=20–66) and 23 (n=19; IQR=12–28) days, respectively. Of 170 returning healthy-travelers to China, 4.7% were asymptomatic carriers (8/170) within 2 weeks, and the IgG and IgM positivity rate was 12.8% (12/94). IgM/IgG-positivity confirmed 3 suspected SARS-CoV-2 cases, despite negative results for SARS-CoV-2 RNA. Compared with other respiratory viral infectious diseases, COVID-19 has fewer asymptomatic carriers, lower antibody response rates, and a longer antibody production duration in recovered patients and the contacted healthy population. This is an indication of the complexity of COVID-19 transmission.

## Introduction

Coronavirus disease 2019 (COVID-19) (Zhou et al., 2020) was officially declared a pandemic and public health emergency of international concern by the World Health Organization, indicating that it may result in substantial morbidity and mortality (Jones, 2020). As of February 16, 2021, more than 109,820,928 confirmed cases of severe acute respiratory syndrome coronavirus 2 (SARS-CoV-2) infections have been reported from 209 countries, including 101,569 cases in China. In total, 2,417,402 patients have died because of COVID-19 (Peeri et al., 2020). However, data on viral load kinetics in confirmed cases and carriers and the production time and distribution of antibodies among patients and the contacted healthy population remain scarce to date (Wu et al., 2020). Currently, there are no data on the proportion of asymptomatic carriers and antibody distribution among the returning healthy travelers from endemic areas. Thus, this study aimed to monitor SARS-CoV-2 viral RNA loads and specific serum-antibodies (immunoglobulin [Ig] G and M) in confirmed patients and asymptomatic carriers among returning healthy travelers.

## Materials and Methods

### Study Subjects and Design

This was a retrospective case–control study on the viral RNA load and antibodies in confirmed COVID-19 patients and asymptomatic carriers. We evaluated all hospitalized COVID-19 patients (n=57) and asymptomatic carriers (n=8) who were admitted to Henan Provincial People’s Hospital between January 2, 2020, and April 29, 2020, and were diagnosed with SARS-CoV-2 infection by positive nucleic acid and antibody tests. In addition, 170 healthy travelers who were returning to China were also evaluated. Patients with negative real-time reverse-transcription polymerase chain reaction (rRT-PCR) results or no SARS-CoV-2-specific IgM and IgG, with a definite diagnosis of other diseases, and with a negative SARS-CoV-2 nucleic acid or antibody test were excluded from this study. Case definitions of confirmed human infection and asymptomatic carriers with SARS-CoV-2 were in accordance with the latest diagnostic criteria (version 7) issued by the Chinese National Health Committee (5). This included patients that had a contact history with confirmed or suspected COVID-19 patients and those that tested positive for SARS-CoV-2 nucleic acid in throat swabs, sputum, or stool samples, or tested positive for IgM/IgG in serum. Further, the patients who had abnormal chest computed tomography (CT) findings and with fever and respiratory symptoms were defined as confirmed cases. Among the patients with a contact history who tested positive for SARS-CoV-2 RNA, those who had normal chest CT findings and no fever or other respiratory symptoms within 14 days of isolation were defined as asymptomatic carriers (Jin et al., 2020).

In total, 65 individuals were evaluated in this study, including 57 confirmed COVID-19 patients and 8 asymptomatic carriers. Among the 57 confirmed COVID-19 patients, 54 patients were local residents and 3 patients who had returned to China from Iran and France were confirmed to have COVID-19 based on IgM or IgG positivity despite negative nucleic acid test results. Notably, 3 patients and 8 asymptomatic carriers were among the 170 returning travelers.

This study was approved by the Institutional Ethics Board of Henan Provincial People’s Hospital (20190050) and was conducted in accordance with the Declaration of Helsinki. The ethics committee waived the requirement for written informed consent for the patients’ participation in this study.

### Diagnosis and Data Collection

Throat swab, sputum, and stool samples for suspected cases were collected for SARS-CoV-2 testing using rRT-PCR assays (Shanghai Zhijiang Biotechnology Ltd., China). Cycle threshold (C_t_) values of ≤44 from the rRT-PCR were considered positive. The duration of the continuous positive C_t_ value was the number of days of viral habitation time in each patient (Padoan et al., 2020). Viral RNA load was presented as RNA copy number of SARS-CoV-2. Ct values of rRT-PCR were converted into RNA copy number of SARS-CoV-2. The RNA copy number was calculated using a reference method as follows. C_t_ values were inversely related to the viral RNA copy number, with C_t_ values of 30.86, 28.68, 24.56, and 21.48, corresponding to 1.5×10^4^, 1.5×10^5^, 1.5×10^6^, and 1.5×10^8^ copies/mL, respectively (Zou et al., 2020). Negative samples were denoted with a C_t_ value of 45, which was under the limit of detection. Additionally, blood specimens for IgM and IgG detection using chemiluminescence assay (Beijing Beier Biotechnology Ltd., China) were considered positive at a cut-off value if the number of antibodies were ≥8 U/mL (Zou et al., 2020). Among the 65 patients, only 37 patients underwent IgM/IgG testing (23 of whom tested positive) because the antibody test was not available in our hospital until 24 February 2020. Information regarding the dates of illness onset, visits to clinical facilities, and hospital admissions were collected from clinical records. The incubation period was defined as the time from exposure to illness onset and was estimated among patients who could provide the exact dates of close contact with individuals who had confirmed or suspected SARS-CoV-2 infection. Throat swab, sputum, and stool samples were collected and tested as the standard point of care diagnostic workup. However, these samples were not collected and tested at the same time. According to the latest Chinese COVID-19 diagnosis and treatment guidelines (version 7, released on March 4, 2020), in suspected patients, whose diagnosis is confirmed by nucleic acid testing, a throat swab is the first sample to be collected, followed by sputum and stool. Additionally, during the patient’s treatment, physicians generally decide on the number and interval of sample collections based on the patient’s response to treatment and recovery status. However, patients are required to have two consecutive negative nucleic acid test results with an interval of >24 h before hospital discharge. Available sequential C_t_ values of rRT-PCR assays and IgM and IgG test results for those included were obtained from the Henan Provincial People’s Hospital laboratory information system. Dates of disease onset, hospitalization and classification of COVID-19 severity were recorded. The date of illness onset was defined as the day when any symptoms were noticed by the patient and later confirmed by a physician. Severity classification was defined using the diagnostic and treatment guidelines for SARS-CoV-2 issued by the Chinese National Health Committee (version 7) (Jin et al., 2020). Patients were defined to have severe COVID-19 if they met one of the following criteria: 1) respiratory distress with respiratory frequency ≥30/min; 2) pulse oximeter oxygen saturation ≤93% at rest; and 3) oxygenation indexes (artery partial pressure of oxygen/inspired oxygen fraction) ≤300 mmHg.

### Statistical Analyses

Continuous variables are summarized as either means and standard deviations if they were normally distributed or as medians with interquartile ranges (IQRs) if they were non-normally distributed. Meanwhile, categorical variables are presented as the percentages of patients in each category. Categorical variables were compared using chi-square or Fisher’s exact tests, and continuous variables were compared using Student’s *t* tests or Mann-Whitney *U* tests, according to their distribution. The Pearson correlation coefficient (*r* value) was used to describe the correlation between continuous variables, including Ct value, and the IgG/M response of patients and asymptomatic carriers. All statistical analyses and graphs were generated and plotted using GraphPad Prism version 8.00 software (GraphPad Software Inc.) or SPSS software version 20.0 (IBM Corp., Armonk, NY, USA). P-values <0.05 were considered statistically significant.

## Results

### Characteristics of the Study Subjects

Of the 57 assessed patients and 8 asymptomatic carriers among 170 healthy traveling returnees, 7 patients required intensive care unit (ICU) admission. Of them, 6 died of severe disease. The remaining 58 patients had mild-to-severe illness or were asymptomatic carriers. Among the 36 severe patients, the mean (standard deviation) age was 60.4 years (16.5), 55.4% were men and 54 (83.1%) patients had at least one coexisting condition. The mean time from admission to symptom onset in severe patients was 11.6 (8.4) days. The mean length of hospitalization of severe patients was 11 (6.6) days, and the average time taken to obtain a positive PCR result was 13.0 (8.3) days. Furthermore, 29 patients had a history of travel to Hubei Province or contact history with confirmed patients, while 3 patients and 8 asymptomatic carriers were among the 170 overseas returnees during the COVID-19 outbreak. **Table 1** shows this information in more detail.

**Table 1:** Demographics and clinical characteristics in confirmed patients as well as carriers of COVID-19^a^.

Characteristics	Patients with severe disease (n = 36) n (%)	Patients with mild disease and carriers (n = 29) n (%)
Age, years, mean (*SD*)	60.4 (16.5)	38.8 (14.4)
Male	16 (45.7)	20 (69.0)
Time of admission from symptom onset, days, mean (SD)	11.6 (8.4)	6.4 (2.2)
Length of hospitalization, days, mean (*SD*)	11 (6.6)	8.8 (4.0)
Time of positive PCR result, days, mean (*SD*)	13.0 (8.3)	4.2 (3.6)
Time of symptom onset, days, mean (*SD*)	27.1 (9.8)	13.3 (2.3)
30-day mortality	6 (17.1)	0 (0)
Hospitalization in ICU	7 (20)	0 (0)
History of travel to Hubei Province during COVID-19 outbreak	15 (41.7)	14 (48.3)
Contact with confirmed patients	17 (47.2)	6 (20.7)
Fever	28 (80)	15 (51.7)
Dry cough	33 (94.3)	18 (62.1)
Fatigue	13 (37.1)	6 (20.7)
Diarrhea	6 (17.1)	10 (34.5)
Shortness of breath	12 (34.3)	1 (3.4)
Expectoration	10 (28.6)	1 (3.4)
COPD	2 (5.7)	1 (3.4)
Diabetes	16 (45.7)	1 (3.4)
Hypertension	10 (28.6)	3 (10.3)
Cardiovascular disease	6 (17.1)	0 (0)
Hepatitis B	1 (2.9)	2 (6.9)
Hypoalbuminemia	8 (22.9)	1 (3.4)

COPD, chronic obstructive pulmonary disease; CT, computed tomography; ICU, intensive care unit; SD, standard deviation.

a Total number of patients with available data.

### Viral RNA Loads in Throat Swab, Sputum, and Stool Samples

**Figure 1:**
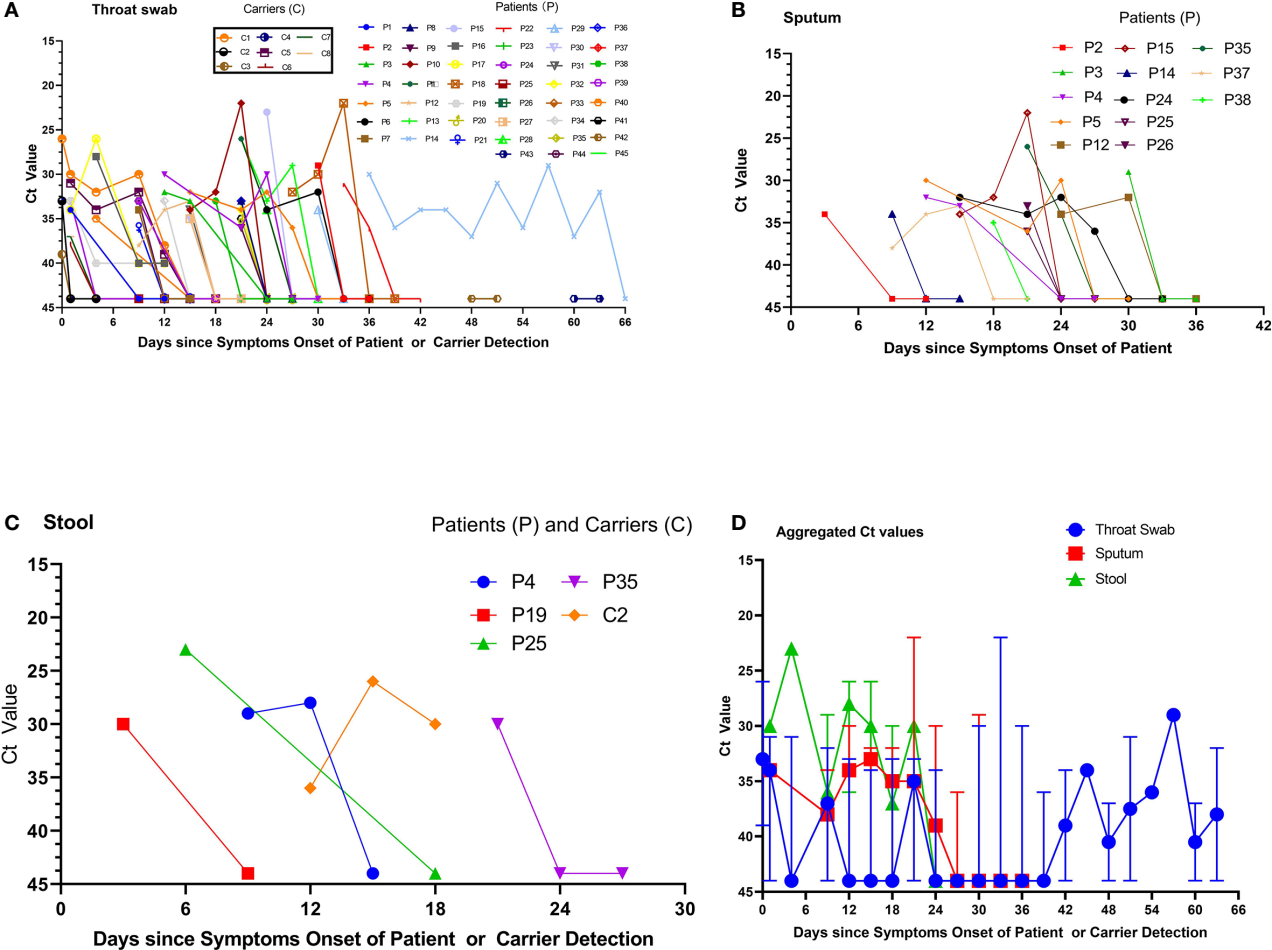
Viral RNA loads detected in throat swabs, sputum samples, and stool specimens obtained from patients infected with severe acute respiratory syndrome coronavirus 2. **(A)** The cycle threshold (C_t_) values of Orf1b in the reverse-transcription polymerase chain reaction (rRT-PCR) assay using throat swab specimens were obtained from the available 45 patients. The labels for the 8 returning asymptomatic carriers are provided in the separate text boxes. **(B)** The C_t_ values of sputum samples and **(C)** the C_t_ values of stool samples from four patients and one carrier. **(D)** The aggregated C_t_ values of Orf1b on the rRT-PCR assay for the 56 cases and 8 carriers, based on the duration from symptom onset to nucleic acid detection.

The C_t_ values were analyzed in a total of 297 samples, including 185 throat swabs, 56 sputum, and 9 stool samples, collected from the 65 patients. The viral RNA loads (inversely related to C_t_ values) detected 3–12 days after symptom onset were higher than those detected after 12 days from symptom onset. The viral RNA loads detected in the sputum, throat, and stool were higher among severely ill patients than among those with mild disease and asymptomatic carriers. The viral RNA loads in the throat swab and sputum samples peaked approximately 3–12 days after symptom onset. The C_t_ values ranged from 34 to 36 (10^5^–10^8^ copies/mL; **Figures 1A, B**) (Zou et al., 2020). Notably, viral RNA was detected in stool samples from 4 symptomatic patients and 1 asymptomatic carrier (**Figure 1C**). The viral RNA loads in stool samples peaked at approximately 6–21 days after symptom onset, with C_t_ values ranging from 23 to 33 (10^5^–10^8^ copies/mL). Among the severely ill patients, the viral RNA loads were higher in the throat swab and sputum samples, and this lasted 33–66 days (**Figure 1D**). Unexpectedly, we found that 8 asymptomatic carriers, on different return flights from Iran, France, Cambodia, and Thailand between March 8, 2020 and April 29, 2020, had a positive nucleic acid result in their oropharyngeal site and intestine tract that lasted 1–8 days, with C_t_ values ranging from 33 to 34 (10^5^–10^8^ copies/mL) (**Figure 1A**).

### Correlation and Comparison of Ct Values Among Sputum, Throat Swab, and Stool Samples

**Figure 2:**
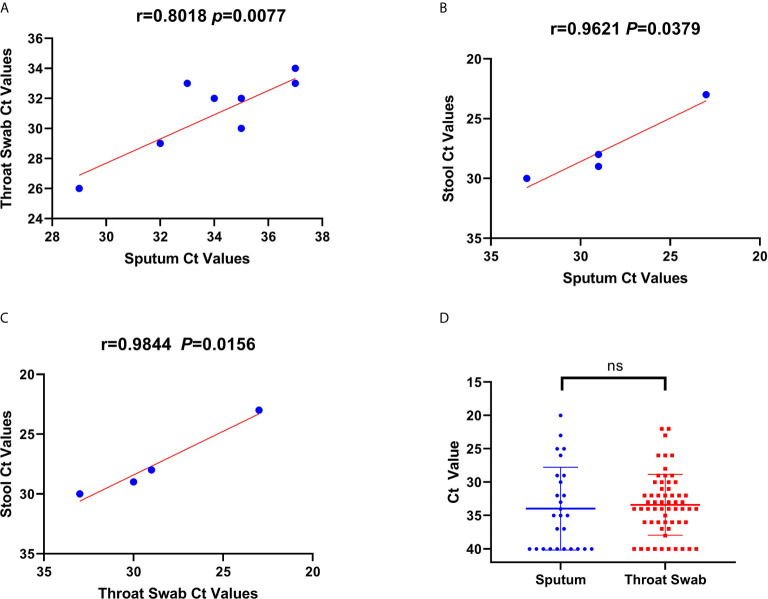
Correlations and comparisons among viral RNA loads detected in throat swabs, sputum samples, and stool samples obtained from patients infected with severe acute respiratory syndrome coronavirus 2. **(A)** The correlation of viral RNA loads between the sputum and throat swab samples. This shows the cycle threshold (C_t_) values of Orf1b in the reverse-transcription polymerase chain reaction assay that were detected from throat swab samples obtained from 56 patients and sputum samples from 13 patients. **(B, C)** The correlation of C_t_ values among throat swab, sputum, and stool samples. There was a positive linear relationship between C_t_ values for the viral RNA loads among the sputum, throat swab, and stool samples obtained from patients. **(D)** The comparison of C_t_ values for viral RNA loads between the throat swab and sputum samples. There was no significant difference in the C_t_ values between sputum and throat swab samples.

Available sequential C_t_ values of every 1-day interval were used to determine the correlation of the viral RNA loads among the throat swab, sputum, and stool samples from the 65 confirmed patients and carriers. The viral RNA loads were significantly correlated between the throat swab and sputum samples (n=28 pairs, R=0.8018, p=0.0088; **Figure 2A**). Similarly, the viral RNA loads were also significantly correlated between the stool and sputum samples (n=8 pairs, R=0.9621, p=0.0389; **Figure 2B**) and between stool and throat swab samples (R=0.98, p=0.0156; **Figure 2C**). Meanwhile, the viral RNA loads and positive PCR duration differed among the sputum, throat swabs, and stool samples. Stool samples had higher viral RNA loads and shorter positive PCR duration than did sputum and throat swab samples (**Figure 1D**). However, there were no significant differences in the viral RNA loads or positive PCR duration between the sputum and throat swab samples from 27 patients that had paired sputum and throat swab samples (**Figures 1D** and **2D**).

### Correlation and Comparison of Viral Duration for Patients by Ward and Severity Classification

**Figure 3:**
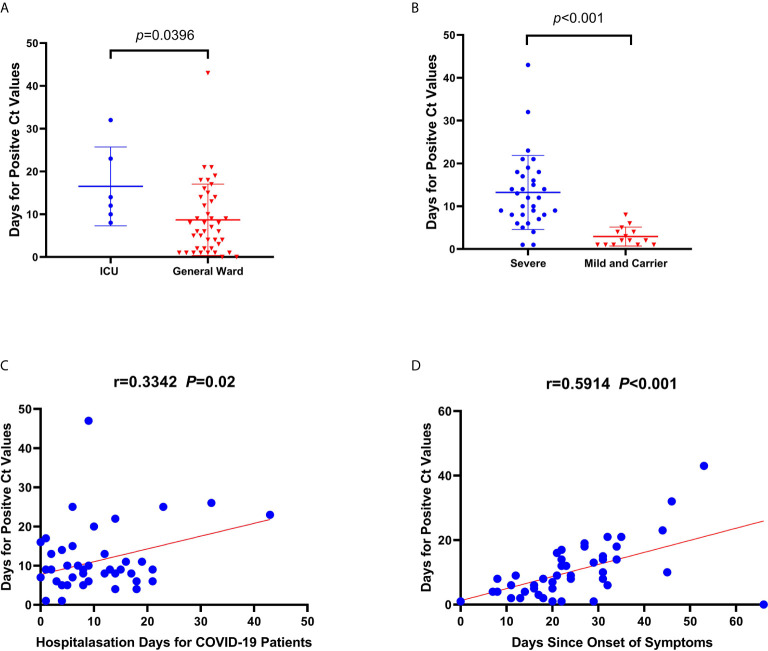
Correlations and comparisons for viral RNA loads detected among coronavirus disease patients admitted to the intensive care unit (ICU) and the general ward, and between severely ill and mildly ill patients and returning carriers with severe acute respiratory syndrome coronavirus 2 infection. **(A)** Comparison of the duration (days) for obtaining positive cycle threshold (C_t_) values since symptom onset between ICU and general ward patients. **(B)** Comparison of the duration (days) for positive C_t_ values between severe and mild patients, and among asymptomatic carriers. **(C, D)** The linear correlations between days for positive C_t_ values and hospitalization days, and days since symptom onset to nucleic acid detection, respectively. There was a significant difference in duration until positive C_t_ values between severe and mild patients, and for asymptomatic carriers. Similarly, the same significant difference in duration was observed for positive C_t_ values between patients from the ICU and the general ward. The duration until a positive C_t_ value in patients was related to hospitalization duration and the duration of symptoms in patients.

To examine viral presence, we compared the duration in days for positive C_t_ values according to the type of ward and severity classification of the patient. The duration for available positive C_t_ values (47 patients and 8 asymptomatic carriers) in the sputum, throat swab, and stool samples was longer for patients in the ICU (n=7) than for those in the general ward (n=46) (median: 13 days [IQR=9.5–25.25 days] vs. median: 6.5 days, [IQR=2–14] p=0.0396, **Figure 3A**). In addition, the duration until available positive C_t_ values were acquired was longer among severe cases (n=30) than those among mild cases and asymptomatic carriers (n=23) (median=12 [IQR=8–18.85 days] vs. median=2 [IQR=1–4.25 days], p<0.001, **Figure 3B**). There was a mutual linear positive relationship among the number of days until a positive C_t_ value, hospitalization days, and days from symptom onset to SARS-CoV-2 detection (**Figures 3C, D**).

### Characteristics of Asymptomatic Carriers and Confirmed COVID-19 Cases Based on Antibodies

**Figure 4:**
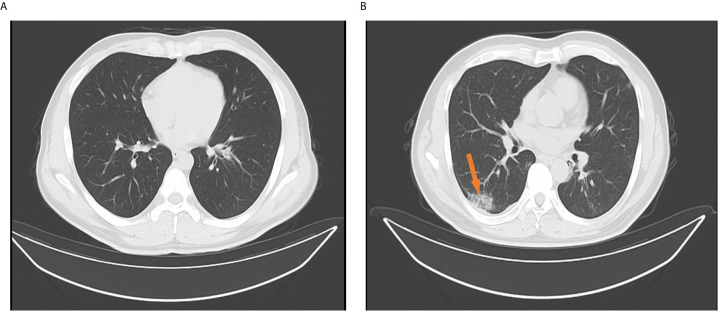
Chest computed tomography findings for confirmed cases and returning carriers with severe acute respiratory syndrome coronavirus-2 infection. **(A)** Normal chest computed tomography characteristics among returning asymptomatic carriers with only positive reverse-transcription polymerase chain reaction (rRT-PCR) results and no signs or symptoms of coronavirus disease (COVID-19) within 14 days. The images were taken the day after hospital admission and after 14 days. **(B)** A solitary rounded ground-glass opacity in the upper lobe (arrow) of the left lung in a confirmed COVID-19 patient with only a positive specific IgM or IgG without sequential positive rRT-PCR assay results. The images were taken when patients were hospitalized or on the next day.

Among the 170 returning healthy travelers, 12 individuals had serum samples that tested positive for SARS-CoV-2 based on specific IgM or IgG antibodies. Further, eight throat swab samples from these individuals were positive for SARS-CoV-2 on rRT-PCR. The positive antibody and nucleic acid results were taken from different individuals among the 170 returning cases. Eight individuals with positive rRT-PCR results had no typical signs or symptoms of SARS-CoV-2, such as fever or cough, within the 14-day mandated quarantine period. Their chest CT scans showed normal imaging features (**Figure 4A**), and their laboratory test results were within normal range (**Table 2**). These 8 (4.7%) individuals were diagnosed as asymptomatic carriers. The IgM and IgG positivity rates were 12.8% (12/94). Among the 56 patients, 3 suspected patients with both positive IgM and IgG results but negative rRT-PCR results were diagnosed with COVID-19 according to the latest diagnostic criteria (version 7) (Jin et al., 2020). These 3 individuals had signs of infection, abnormal laboratory test results (**Table 1**), and abnormal findings on CT (**Figure 4B**).

**Table 2:** Laboratory and physical examination results for the eight asymptomatic carriers and three confirmed cases with severe acute respiratory syndrome coronavirus 2 diagnosed according to a positive antibody test.

Parameter	Reference Range	Carrier 1	Carrier 2	Carrier 3	Carrier4	Carrier 5	Carrier 6	Carrier 7	Carrier 8	Patient 1*	Patient 2*	Patient 3*
PCR	–	+	+	+	+	+	+	+	+	–	–	–
IgM	–	–	–	–	–	–	+	+	+	+	+	+
IgG	–	–	–	–	–	–	–	–	–	+	+	–
C-reactive protein (mg/L)	0.0–10	0.6	0	0.3	8.8	2.5	2	0.5	1.5	35.4	48.6	3.3
Eosinophils (109/L)	0.02–0.52	0.05	0.02	0.1	0.06	0.38	0.13	0.04	0.12	0.02	0.01	0
Lymphocytes (109/L)	1.1–3.2	1.8	1.55	1.2	2.22	2.48	2.55	0.91	1.55	1.38	3.38	1.55
Neutrophils (109/L)	1.8–6.3	3.48	5.21	2.26	2.2	2.25	5.93	2.03	1.86	3.01	4.11	4.35
White blood cell count, (109/L)	3.9–9.9	5.5	6.8	4.1	3.4	5.5	9.1	3.4	3.9	5	8.1	6.2
D-dimer (mg/L)	0–0.5	0.19	0.19	<0.19	0.21	0.19	0.19	0.19	0.19	0.2	0.83	0.28
Procalcitonin (µg/L)	0–0.25	0.04	0.08	0.05	0.03	0.06	0.05	0.09	0.14	0.12	0.28	0.03
Chest CT	–	–	–	–	–	–	–	–	–	Abnormality	Abnormality	Abnormality
Signs and symptoms	–	–	–	–	–		–	–	–	Fever, Cough	Fever, Cough	Fever, Cough

*Two sequential negative PCR results for severe acute respiratory syndrome coronavirus 2 with an interval of more than 24 hours. +: positive; -: negative. PCR, polymerase chain reaction; CT, computed tomography; Ig, immunoglobulin.

### Distribution of IgM and IgG Antibodies Among Confirmed Patients and Carriers

Among the 29 antibody-positive patients, only 55.2% (16/29) and 58.6% (17/29) were IgG and IgM positive, respectively, within 66 days from symptom onset. Meanwhile, 3 of the 8 asymptomatic carriers had positive serum IgM and IgG results. The sensitivity of rRT-PCR for COVID-19 diagnosis in the 66 days following symptom onset was 94.6% (53/56), which was significantly greater than the 62.2% (23/37) sensitivity of the antibody test (χ2 = 16.95, p<0.001). Of the 29 confirmed patients and 8 asymptomatic carriers among the 170 healthy traveling returnees, 20 patients had positive antibody test results during the 66 days from symptom onset and 3 asymptomatic carriers showed IgM antibody response within 14 days. The median duration from symptom onset to IgG response was 30 days (IQR = 20–66 days), while it was only 23 days (IQR=12–28 days) for IgM-positive patients and asymptomatic carriers.

### Comparison of the Duration to Positive IgG and IgM Results and Positive Ct Value

**Figure 5:**
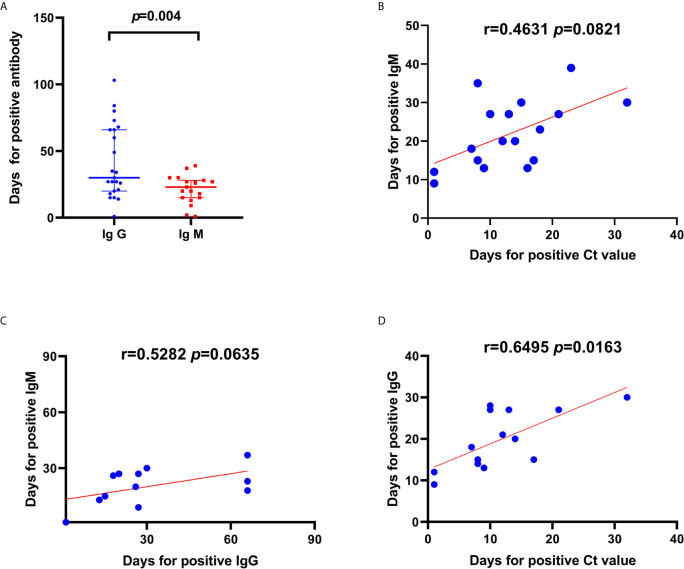
Correlations and comparisons in duration to positive IgG and IgM result and duration until a positive cycle threshold (C_t_) value was detected from patients with severe acute respiratory syndrome coronavirus-2 (SARS-CoV-2) infection. **(A)** Comparison in duration (days) until a positive antibody test since symptom onset between IgG and IgM. **(B)** Linear correlations between duration until positive C_t_ values and duration until positive IgM. Linear correlations between duration until C_t_ value positivity and IgM **(C)** and IgG **(D)** positivity. There was a significant difference in the duration until C_t_ value positivity between IgM and IgG positivity. Moreover, the duration until C_t_ value positivity was correlated to the duration until IgG positivity in patients with SARS-CoV-2 infection.

We compared the duration until positive IgM and IgG results were detected among the available 29 patients and 8 asymptomatic carriers and found a significant difference in the duration (p=0.004, **Figure 5A**). However, there was no significant difference in the duration until a positive IgM result and a positive C_t_ value were obtained (**Figures 5B, C**). Moreover, the duration until a positive C_t_ value was obtained was correlated with the duration to IgG response onset (R=0.6495, p=0.0163; **Figure 5D**). The IgM or IgG positivity rate was higher among severely ill patients than among mild cases and asymptomatic carriers: 71.4% (20/28) vs. 50% (5/10) (χ^2^ = 8.828, p=0.002).

## Discussion

There are currently limited data on the proportion of asymptomatic carriers and antibody distribution among the returning healthy travelers from endemic areas. In this study, asymptomatic infections accounted for 4.7% (8/170) of the returning cases among the healthy population, and the antibody production rate was 12.8% (12/94). This study presents the viral RNA load kinetics and distribution of antibodies in patients and asymptomatic COVID-19 carriers in central China. We speculated that during the 2 weeks following disease onset, disease transmissibility was higher. With recovery onset, the production rate for the antibody response increased to 62.2% (23/37) at 66 days after symptom onset. These data indicate that compared with other known respiratory viral infectious diseases such as hand-foot-and-mouth disease, mumps, and rubella, this emerging infectious disease caused by SARS-CoV-2 has fewer asymptomatic carriers and generates a lower antibody response rate among healthy contacts. Moreover, a longer duration is required for antibody production among both recovered patients and healthy contacts. Further, it is unclear whether the IgG antibody is protective, indicating the complexity of COVID-19 transmission. Therefore, we believe that this new virus may have just started spreading from animals to humans and it may persist in humans for a long time. To the best of our best knowledge, this is the first report of 3 confirmed COVID-19 cases detected using specific IgM or IgG antibody tests from serum specimens with negative rRT-PCR results from throat swab samples among Chinese nationals who returned from endemic countries. Moreover, this is the first report of 8 (4.7%) asymptomatic carriers diagnosed 2 weeks after returning to China among the same population.

A positive IgM result indicates a recent infection with SARS-CoV-2. Additionally, IgG-positive results indicate that the body has begun to establish an immune defense. By testing patients and carriers for IgM and IgG antibodies and identifying time points at which they start producing antibodies, it is possible to monitor the extent to which COVID-19 spreads and the infection duration (Erensoy, 2020). A previous report showed that the rRT-PCR assays could be used to test throat swab samples to detect asymptomatic carriers with a travel history who later transmitted the infection to their contacts (Kannan et al., 2020; Lai et al., 2020). To better manage asymptomatic infections, the Chinese government has stipulated that from April 1, 2020, health authorities should report the daily number of new cases and outcomes of asymptomatic carriers nationwide. On February 18, 2021, there were 338 cases of asymptomatic carriers in China. Among them, 282 were returning asymptomatic carriers. However, the proportion of asymptomatic carriers and rate of antibody production among healthy contacts are unknown. Our findings reinforce that for identification and contact screening of individuals traveling from epidemic countries, it is important to conduct joint detection for SARS-CoV-2 using respiratory samples for nucleic acid testing and blood samples for IgM or IgG antibody testing (Bai et al., 2020).

Consistent with the findings in previous studies, the virus was detected in stool specimens in this study in addition to samples from the upper respiratory tract (Chen et al., 2020; Won et al., 2020). Diagnostic and treatment guidelines recommend detection of SARS-CoV-2 *via* throat swabs using nucleic acid testing (Jin et al., 2020). A stool sample for nucleic acid testing or a blood sample for specific IgM or IgG antibody detection should be obtained from patients highly suspected of COVID-19 but with continuously negative nucleic acid test results from throat swabs.

Unlike SARS-CoV and Middle East respiratory syndrome coronavirus infection (Chafekar and Fielding, 2018; Xu K. et al., 2020), the SARS-CoV-2 viral RNA load is highest during the early phase of the illness then continues to decrease until the end of the second week. In severe cases, the high viral RNA load can last up to 2 months. The duration of the virus infection is positively correlated with the disease severity and symptom duration, suggesting that we should detect, diagnose, and isolate the patients as early as possible to prevent community transmission and mortality.

This study has some limitations. First, the patients may not be representative of the general population of COVID-19 patients in China (Fan et al., 2020; Wu and McGoogan, 2020). Second, we cannot estimate the time point that these patients were exposed to the virus and when viral shedding *via* respiratory secretions and stool started. Third, the virus was not cultured from respiratory secretions and stool specimens because we do not have a professional Bio-safety Level 3 laboratory in our hospital (Xu X. et al., 2020). Finally, there were no sequential IgM or IgG antibody distribution results available for the SARS-CoV-2 infected patients throughout the duration of their illness. The IgG and IgM positivity rates only accounted for 51.4% and 62.6% of the 29 confirmed patients and 8 carriers within 66 days following symptom onset, respectively. The IgM antibody is known to be produced in the early stages of an infectious disease, whereas the IgG antibody is produced during the recovery period (Kam et al., 2020; To et al., 2020). We found that the median duration from symptom onset to IgG and IgM positivity was 30 and 23 days, respectively. This indicated that in COVID-19, initiation of IgG antibody production is longer than that for IgM production at 30 days versus earlier than 23 days. More samples should further be observed to confirm the phenomenon (Amanat et al., 2020).

In conclusion, we found that there are fewer asymptomatic COVID-19 carriers among the returning healthy travelers. Additionally, there are lower rates of antibodies produced among recovered patients and the contacted healthy population. These findings indicate the complexity of COVID-19 transmission and suggest that this infectious disease is likely to occur in humans for a long time.

## Data Availability Statement

The original contributions presented in the study are included in the article/supplementary material. Further inquiries can be directed to the corresponding author.

## Ethics Statement

The studies involving human participants were reviewed and approved by the Institutional Ethics Board of Henan Provincial People’s Hospital (20190050). The ethics committee waived the requirement of written informed consent for participation.

## Author Contributions

YY and HW designed the study, analyzed the data and prepared the manuscript. JinZ and WL contributed to the collection and interpretation of the laboratory and clinical data. NJ and JX analyzed the antibody data of patients and carriers. GL, YL, SW, YW, and LL were involved in the project management and organizational work. BM and JiaZ collected data and EF reviewed the manuscript. All authors contributed to the article and approved the submitted version.

## Funding

This work was supported by the Henan Provincial Key Programs in Science and Technology (CXJD2019002, 202102310355 and 182102310098) and the Joint Program of Medical Science and Technology Research of Henan Province (LHGJ20190611).

## Conflict of Interest

The authors declare that the research was conducted in the absence of any commercial or financial relationships that could be construed as a potential conflict of interest.
